# A New Blow-Pipe

**Published:** 1866-01

**Authors:** 


A New Blow-Pipe.—A novel blow-pipe is thus described: "Hendy's blow-pipe consists of an ordinary blow-pipe nozzle supplied from an india-rubber bag. The main portion of the blow-pipe has a flexible mouth-piece connected with the bag by a joint, at which a valve is placed, which is opened when the operator blows, and closes immediately when he ceases. By this arrangement the little bag or bladder is readily filled at a single breath, and with very little exertion.

When so filled, a continuous current of air is forced from the nozzle of the pipe by the mere contractive force of the bag. This is easily turned in any direction as required, by means of the flexible tube to which it is attached."

